# Supplementary material for: An Efficient and Flexible Method for Deconvoluting Bulk RNA-Seq Data with Single-Cell RNA-Seq Data
Source: Cells. 2019 Sep 27;8(10):1161. doi: 10.3390/cells8101161 (PMC6830085; doi:10.3390/cells8101161)

Supplementary Text: An Efficient and Flexible Method for Deconvoluting Bulk RNA-Seq Data with Single-Cell RNA-Seq Data

Xifang Sun [1], Shiquan Sun [2,3] and Sheng Yang [4,*]

[1] Department of Mathematics, School of Science, Xi'an Shiyou University, 710065 Xi'an, Shaanxi, China
[2] School of Computer Science, Northwestern Polytechnical University, 710072 Xi'an, Shaanxi, China
[3] Department of Biostatistics, University of Michigan, Ann Arbor, MI 48109, USA
[4] Department of Biostatistics, School of Public Health, Nanjing Medical University, 211166 Nanjing, Jiangsu, China
* Correspondence: yangsheng@njmu.edu.cn; Tel.: +86 2586868241

MOMF Deconvolution Method

1. Model

We directly model the count nature of both scRNA-seq data and bulk RNA-seq data through Poisson models to jointly deconvolute bulk RNA-seq data. Specifically, we denote the expression count matrix for scRNA-seq data as $\boldsymbol{X}$ and denote the expression count matrix for bulk RNA-seq data as $\mathbf{Y}$.

For bulk RNA-seq data, we consider

$$\boldsymbol{Y}_{ij} \sim Poisson\left(\boldsymbol{\mu}_{ij}^{y}\right), i = 1,2,\cdots,n_y; j = 1,2,\cdots,p, \tag{1}$$

where $\boldsymbol{Y}_{ij}$ is the number of reads that measure the gene expression levels for j'th gene and i'th individual; n_y is the number of individuals; $\boldsymbol{\mu}_{ij}^{y}$ is an unknown Poisson rate parameter that represents the underlying mean gene expression level for the i'th individual and j'th gene; and p is the number of genes; $Poisson(\cdot)$ represents the Poisson distribution.

For scRNA-seq data, we consider

$$\boldsymbol{X}_{kj} \sim Poisson\left(\boldsymbol{\mu}_{kj}^{x}\right), k = 1,2,\cdots,n_x; j = 1,2,\cdots,p\,, \tag{2}$$

where $\boldsymbol{X}_{kj}$ is the number of reads that measure the gene expression level for j'th gene and k'th cell; n_x is the number of cells; $\boldsymbol{\mu}_{kj}^{x}$ is an unknown Poisson rate parameter that represents the underlying gene expression level for the i'th cell and j'th gene; and p is the number of genes; $Poisson(\cdot)$ represents the Poisson distribution.

In above models, we further decompose the unknown parameters $\boldsymbol{\mu}_{ij}^{y}$ and $\boldsymbol{\mu}_{kj}^{x}$ into two low-dimension matrices, i.e.,

$$\boldsymbol{\mu}_{ij}^{y} = \sum\nolimits_{c=1}^{C} \boldsymbol{\Psi}_{ic}\boldsymbol{W}_{cj} + \boldsymbol{E}_{ij}^{y}, i = 1,2,\cdots,n_y; j = 1,2,\cdots,p, \tag{3}$$

where $\boldsymbol{\Psi}_{ic}$ is the cell type specific proportion for the i'th individual and c'th cell type; C is the number of cell types.

$$\boldsymbol{\mu}_{kj}^{x} = \sum\nolimits_{c=1}^{C} \boldsymbol{\Lambda}_{kc}\boldsymbol{W}_{cj} + \boldsymbol{E}_{kj}^{x}, k = 1,2,\cdots,n_x; j = 1,2,\cdots,p, \tag{4}$$

where $\boldsymbol{\Lambda}_{kc}$ is the low-dimension structure for the k'th cell and c'th cell type; C is the number of cell type; the parameter $\boldsymbol{W}_{cj}$ is the element in the factor loading matrix that represents the underlying true cell-type specific gene expression level; the factor loading matrix $\boldsymbol{W}$ is shared between bulk RNA-seq and scRNA-seq data, allowing us to jointly model both data types and bypassing the estimation uncertainty inevitably occur in previous deconvolution methods; $\boldsymbol{E}_{ij}^{y}$ and $\boldsymbol{E}_{kj}^{x}$ are the residual terms that account for over-dispersion commonly observed in sequencing studies for bulk RNA-seq data and scRNA-seq data,

respectively.

2. ADMM algorithm

To utilize the ADMM algorithm, we first construct the objective function:

$$\mathcal{L} = D(\boldsymbol{Y}|\boldsymbol{\mu}^y) + \boldsymbol{Tr}(\boldsymbol{U}^y(\boldsymbol{\mu}^y - \boldsymbol{\Psi W})^T) + \frac{\rho}{2}||\boldsymbol{\mu}^y - \boldsymbol{\Psi W}||_F^2 + \boldsymbol{Tr}(\boldsymbol{U}^{\Psi}(\boldsymbol{\Psi} - \boldsymbol{\Psi}_+)^T) +$$
$$\frac{\rho}{2}||\boldsymbol{\Psi} - \boldsymbol{\Psi}_+||_F^2 + D(\boldsymbol{X}|\boldsymbol{\mu}^x) + \boldsymbol{Tr}(\boldsymbol{U}^x(\boldsymbol{\mu}^x - \boldsymbol{\Lambda W})^T) + \frac{\rho}{2}||\boldsymbol{\mu}^x - \boldsymbol{\Lambda W}||_F^2 +$$
$$\boldsymbol{Tr}(\boldsymbol{U}^{\Lambda}(\boldsymbol{\Lambda} - \boldsymbol{\Lambda}_+)^T) + \frac{\rho}{2}||\boldsymbol{\Lambda} - \boldsymbol{\Lambda}_+||_F^2 + \boldsymbol{Tr}(\boldsymbol{U}^W(\boldsymbol{W} - \boldsymbol{H})^T) + \frac{\rho}{2}||\boldsymbol{W} - \boldsymbol{H}||_F^2, \quad (5)$$

where $D(y|x) = ylog\left(\frac{y}{x}\right) - y + x$ is the Kullback-Leibler (KL) divergence; $\boldsymbol{U}^y$, $\boldsymbol{U}^x$, $\boldsymbol{U}^{\Psi}$, $\boldsymbol{U}^{\Lambda}$ and $\boldsymbol{U}^W$ are element-wise coefficients; $\boldsymbol{\Psi}_+$ and $\boldsymbol{\Lambda}_+$ are the non-negative matrix for $\boldsymbol{\Psi}$ and $\boldsymbol{\Lambda}$, respectively; ρ is the penalty parameter; $\boldsymbol{H}$ is reference gene expression panel; $\boldsymbol{W}$ is underlying true gene expression panel; $\boldsymbol{Tr}(\cdot)$ denotes the trace of a matrix. .

2.1 Update $\boldsymbol{\mu}^x$ and $\boldsymbol{\mu}^y$

When $\beta = 1$ and $D(y|x) = ylog\left(\frac{y}{x}\right) - y + x$, we update $\boldsymbol{\mu}^x$ and $\boldsymbol{\mu}^y$, respectively.

For bulk RNA-seq data, we consider

$$D_{\beta=1}(\boldsymbol{Y}|\boldsymbol{\mu}^y) = \boldsymbol{Y}log\frac{\boldsymbol{Y}}{\boldsymbol{\mu}^y} - \boldsymbol{Y} + \boldsymbol{\mu}^y$$

$$\frac{\partial D_{\beta=1}(\boldsymbol{Y})}{\partial \boldsymbol{\mu}^y} = -\frac{\boldsymbol{Y}}{\boldsymbol{\mu}^y} + 1 + \boldsymbol{U}^y + \rho(\boldsymbol{\mu}^y - \boldsymbol{\Psi W}) = 0$$

$$\boldsymbol{\mu}_{ij}^y = \frac{\rho \boldsymbol{\Psi}_{ij}\boldsymbol{W}_{cj} - \boldsymbol{U}_{ij}^y - 1 + \sqrt{\left(\rho \boldsymbol{\Psi}_{ij}\boldsymbol{W}_{cj} - \boldsymbol{U}_{ij}^y - 1\right)^2 + 4\rho \boldsymbol{Y}_{ij}}}{2\rho} \quad (6)$$

For scRNA-seq data, we consider

$$D_{\beta=1}(\mathbf{X}|\boldsymbol{\mu}^x) = \mathbf{X}\log\frac{\mathbf{X}}{\boldsymbol{\mu}^x} - \mathbf{X} + \boldsymbol{\mu}^x$$

$$\frac{\partial D_{\beta=1}(\mathbf{X})}{\partial \boldsymbol{\mu}^x} = -\frac{\mathbf{X}}{\boldsymbol{\mu}^x} + 1 + \mathbf{U}^x + \rho(\boldsymbol{\mu}^x - \mathbf{\Lambda W}) = 0$$

$$\boldsymbol{\mu}_{kj}^x = \frac{\rho \boldsymbol{\Lambda}_{kj}\boldsymbol{W}_{cj} - \boldsymbol{U}_{kj}^x - 1 + \sqrt{\left(\rho \boldsymbol{\Lambda}_{kj}\boldsymbol{W}_{cj} - \boldsymbol{U}_{kj}^x - 1\right)^2 + 4\rho \boldsymbol{X}_{kj}}}{2\rho} \quad (7)$$

2.2 Update $\boldsymbol{\Psi}$ and $\boldsymbol{\Lambda}$

Taking the derivative of $\mathcal{L}$ with respect to $\boldsymbol{\Psi}_{ij}$ and $\boldsymbol{\Lambda}_{ij}$, we have

$$\frac{\partial \mathcal{L}_\beta}{\partial \boldsymbol{\Psi}} = \quad -\boldsymbol{U}^y\boldsymbol{W} - \rho[\boldsymbol{Y} - \boldsymbol{\Psi}\boldsymbol{W}]\boldsymbol{W}^{\boldsymbol{T}} + \boldsymbol{U}^{\Psi} + \rho(\boldsymbol{\Psi} - \boldsymbol{\Psi}_+) = 0$$

$$\boldsymbol{\Psi} = (\boldsymbol{W}\boldsymbol{W}^{\boldsymbol{T}} + \boldsymbol{I})^{-1}\left\{\boldsymbol{Y}\boldsymbol{W}^{\boldsymbol{T}} + \boldsymbol{\Psi}_+ + \frac{1}{\rho}[\boldsymbol{U}^y\boldsymbol{W}^{\boldsymbol{T}} - \boldsymbol{U}^{\Psi}]\right\} \tag{8}$$

$$\frac{\partial \mathcal{L}_\beta}{\partial \boldsymbol{\Lambda}} = \quad -\boldsymbol{U}^x\boldsymbol{W} - \rho[\boldsymbol{X} - \boldsymbol{\Psi}\boldsymbol{W}]\boldsymbol{W}^{\boldsymbol{T}} + \boldsymbol{U}^{\Lambda} + \rho(\boldsymbol{\Lambda} - \boldsymbol{\Lambda}_+) = 0$$

$$\boldsymbol{\Lambda} = (\boldsymbol{W}\boldsymbol{W}^{\boldsymbol{T}} + \boldsymbol{I})^{-1}\left\{\boldsymbol{X}\boldsymbol{W}^{\boldsymbol{T}} + \boldsymbol{\Lambda}_+ + \frac{1}{\rho}[\boldsymbol{U}^x\boldsymbol{W}^{\boldsymbol{T}} - \boldsymbol{U}^{\Lambda}]\right\} \tag{9}$$

2.3 Update $\boldsymbol{W}$

Taking the derivative of $\mathcal{L}$ with respect to $\mathbf{W}$, we have

$$\frac{\partial \mathcal{L}_\beta}{\partial \boldsymbol{W}} = \{-\boldsymbol{\Psi}^T\boldsymbol{U}^y - \rho\boldsymbol{\Psi}^T[\boldsymbol{Y} - \boldsymbol{\Psi}\boldsymbol{W}] - \boldsymbol{\Lambda}^{\boldsymbol{T}}\boldsymbol{U}^x - \rho\boldsymbol{\Lambda}^{\boldsymbol{T}}[\boldsymbol{X} - \boldsymbol{\Lambda}\boldsymbol{W}]\} + \boldsymbol{U}^W + \rho(\boldsymbol{W} - \boldsymbol{W}_+) = \mathbf{0}$$

$$\boldsymbol{W} = [\boldsymbol{\Psi}^T\boldsymbol{\Psi} + \boldsymbol{\Lambda}^{\boldsymbol{T}}\boldsymbol{\Lambda} + \boldsymbol{I}]^{-1}\left\{\boldsymbol{\Psi}^T\boldsymbol{Y} + \boldsymbol{\Lambda}^{\boldsymbol{T}}\boldsymbol{X} + \boldsymbol{W}_+ + \frac{1}{\rho}[\boldsymbol{\Psi}^T\boldsymbol{U}^y + \boldsymbol{\Lambda}^{\boldsymbol{T}}\boldsymbol{U}^x - \boldsymbol{U}^W]\right\} \tag{10}$$

2.4 Updating $\boldsymbol{\Psi}_+$ and $\boldsymbol{\Lambda}_+$

$$\boldsymbol{\Psi}_+ = max\left(\boldsymbol{\Psi} + \frac{1}{\rho}\boldsymbol{U}^y, \mathbf{0}\right), \boldsymbol{\Lambda}_+ = max\left(\boldsymbol{\Lambda} + \frac{1}{\rho}\boldsymbol{U}^x, \mathbf{0}\right) \tag{11}$$

2.5 Updating $\boldsymbol{U}^y$, $\boldsymbol{U}^x$ and $\boldsymbol{U}^W$

$$\boldsymbol{U}^y \leftarrow \boldsymbol{U}^y + \rho(\boldsymbol{\mu}^y - \boldsymbol{\Psi}\boldsymbol{W}), \boldsymbol{U}^x \leftarrow \boldsymbol{U}^x + \rho(\boldsymbol{\mu}^x - \boldsymbol{\Lambda}\boldsymbol{W}), \boldsymbol{U}^W \leftarrow \boldsymbol{U}^W + \rho(\boldsymbol{W} - \boldsymbol{H}) \tag{12}$$

Supplementary Figure 1. The correlation of cell type proportion estimated by MOMF with two independent runs on CRC data. The scatter plot shows the robustness of MOMF. The square of correlation coefficient (R^2) of the cell type proportion matrix is displayed within the scatter plot.

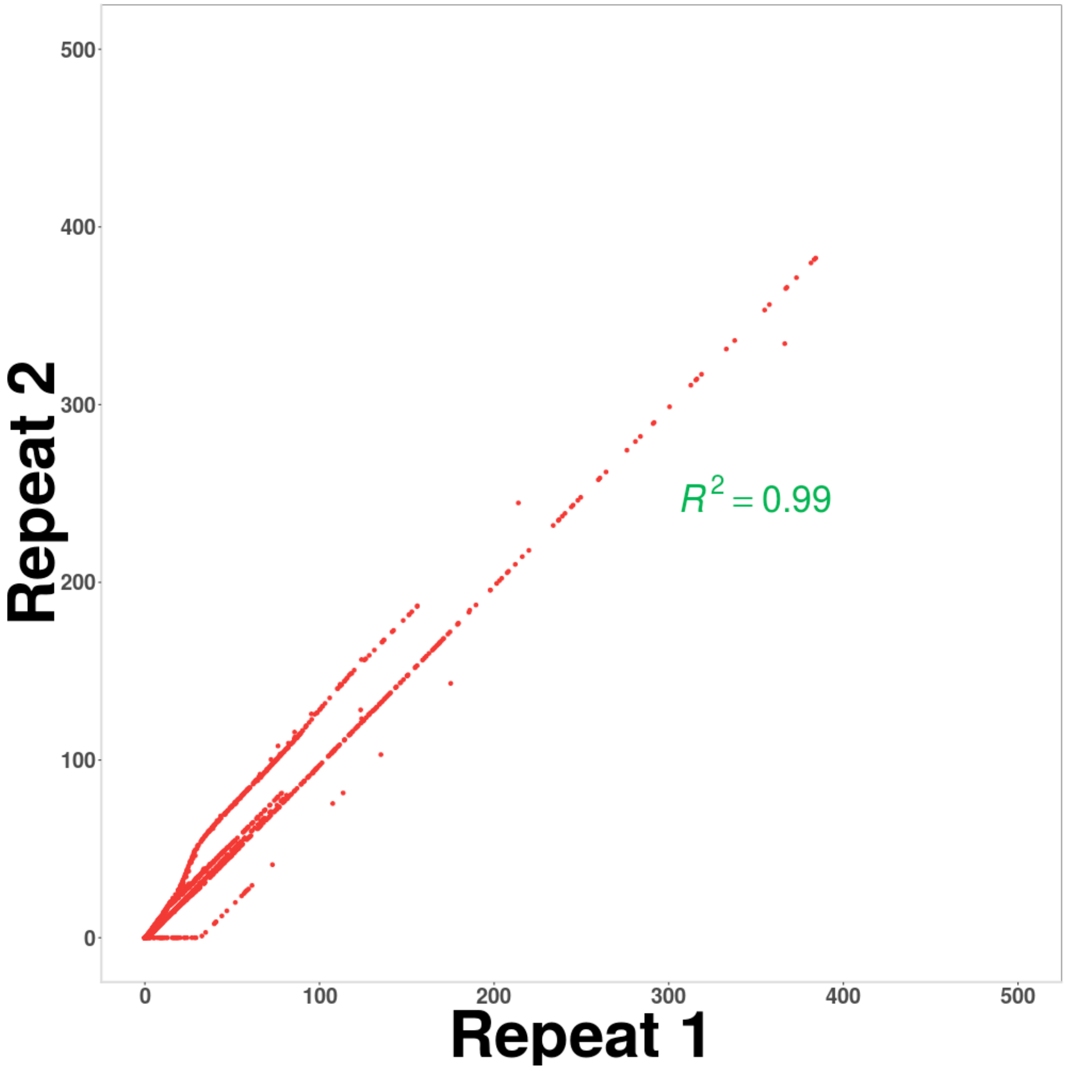

Supplementary Figure 2. An example to show the distortion of normalized gene expression caused by logarithm transformation. We used the histogram of gene expression for gene ENSG00000180725 from CRC scRNA-seq data to show the artificial difference from the logarithm transformation. (**A**) the histogram of raw counts of gene ENSG00000180725. (**B**) the histogram of gene expression after CPM normalization. (**C**) the histogram of gene expression after $\log_2(CPM + 1)$ normalization.

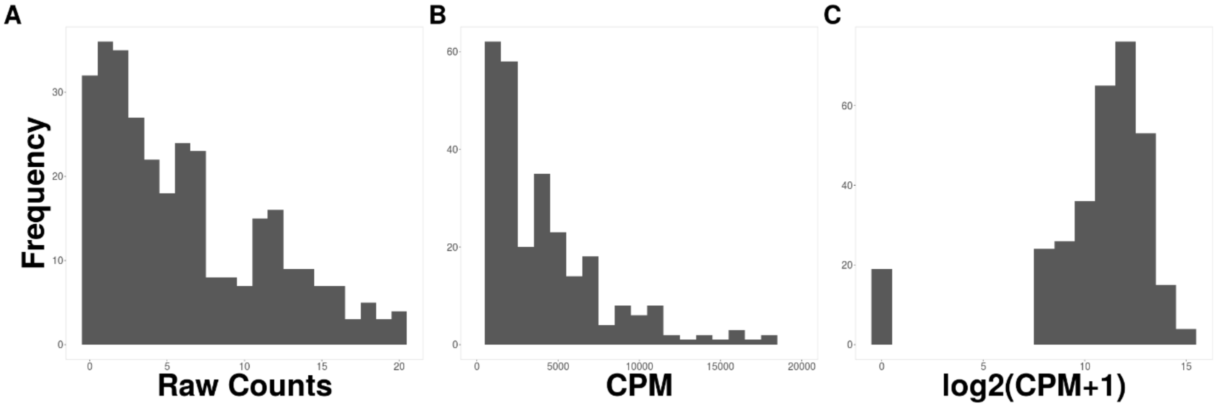

Supplementary Figure 3. Simulation results with 2 cell types. (**A**) The scatter plot of ground truth and cell type proportion estimated by MOMF; (**B**) The boxplot to show the difference between ground truth and cell type proportion estimated by MOMF (**C**) The scatter plot of ground truth and cell type proportion estimated by MuSiC; (**D**) The boxplot to show the difference between ground truth and cell type proportion estimated by MuSiC (**E**) The scatter plot of ground truth and cell type proportion estimated by CIBERSORT; (**F**) The boxplot to show the difference between ground truth and cell type proportion estimated by CIBERSORT. R: Pearson correlation.

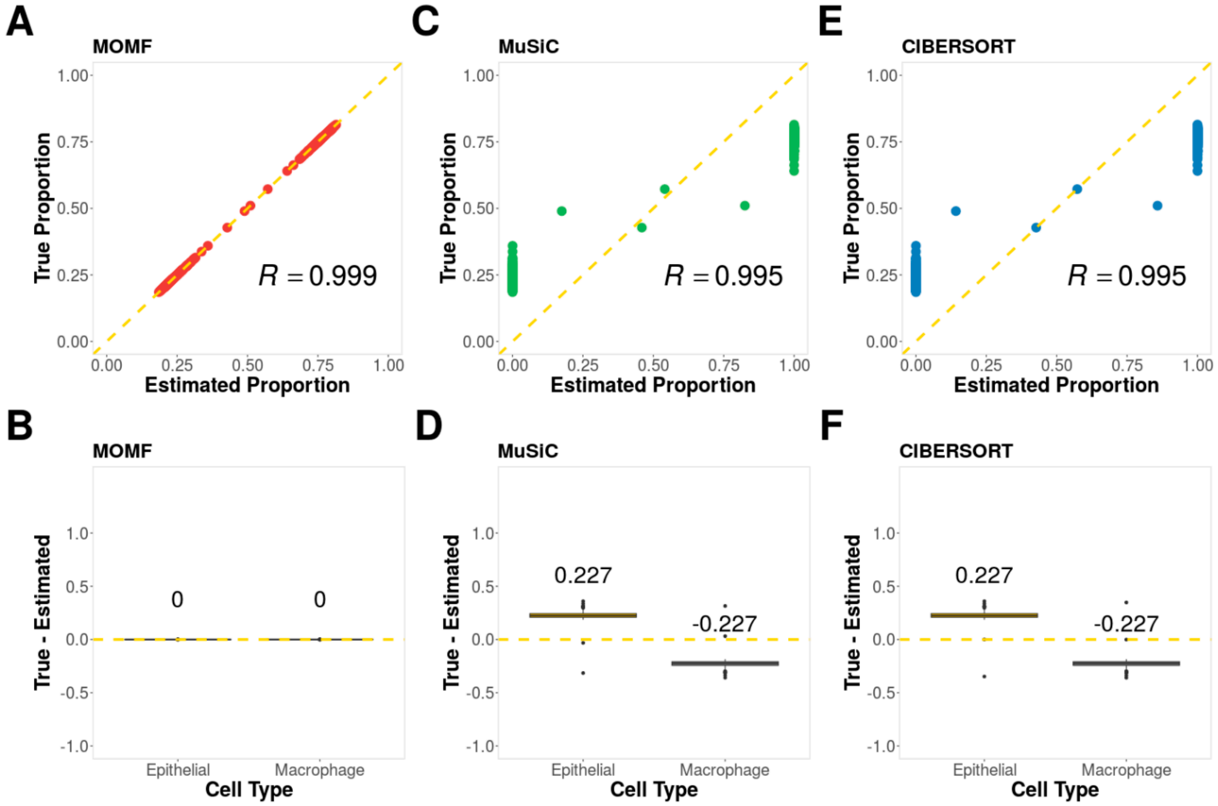

Supplementary Figure 4. Simulation results with 5 cell types. (**A**) The scatter plot of ground truth and cell type proportion estimated by MOMF; (**B**) The boxplot to show the difference between ground truth and cell type proportion estimated by MOMF (**C**) The scatter plot of ground truth and cell type proportion estimated by MuSiC; (**D**) The boxplot to show the difference between ground truth and cell type proportion estimated by MuSiC (**E**) The scatter plot of ground truth and cell type proportion estimated by CIBERSORT; (**F**) The boxplot to show the difference between ground truth and cell type proportion estimated by CIBERSORT. R: Pearson correlation.

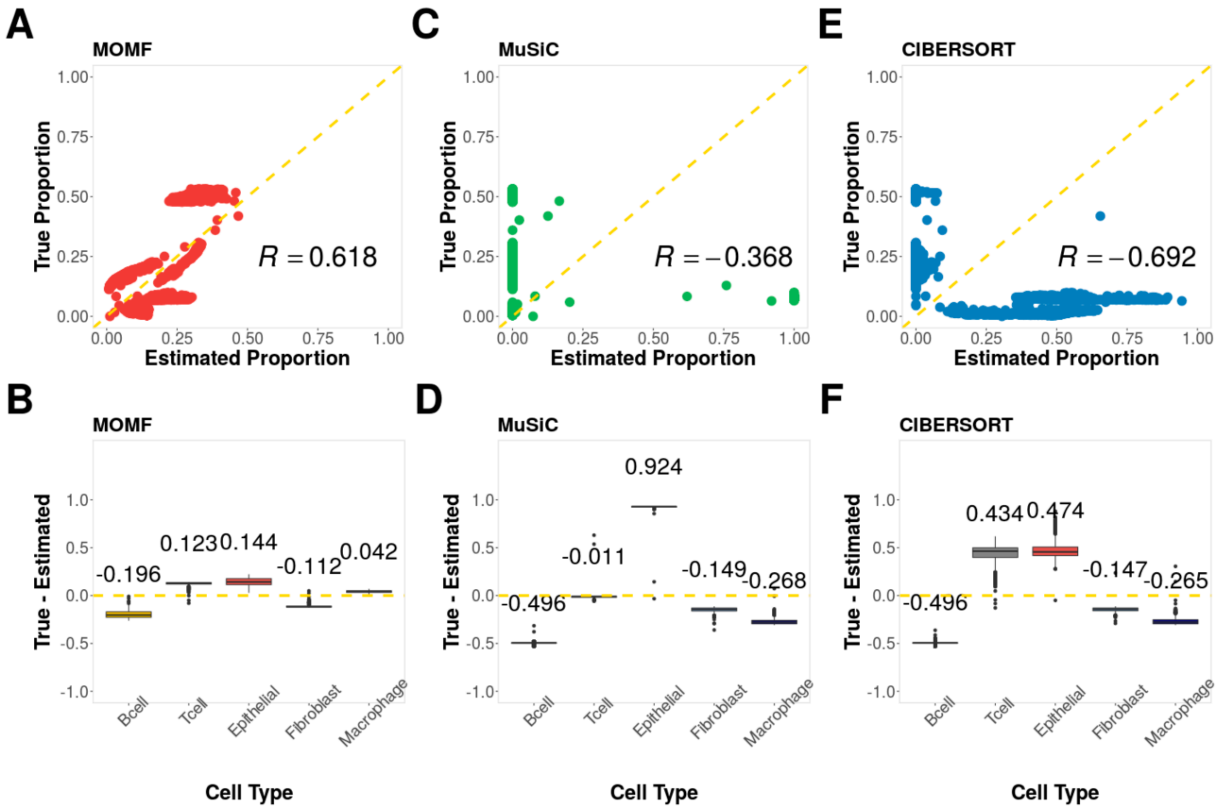

Supplement: Supplementary file 1 [file cells-08-01161-s001.pdf]
